# Multifocal Aspergillus endocarditis: Comprehensive imaging of extensive cardiac involvement in a transplant recipient

**DOI:** 10.21542/gcsp.2025.65

**Published:** 2025-12-31

**Authors:** Nikola Dobrilovic, Athena Poppas, Arun Singh

**Affiliations:** 1 Division of Cardiothoracic Surgery, Rhode Island Hospital, Brown University, Providence, Rhode Island 02903, USA; 2 Division of Cardiology, Rhode Island Hospital, Brown University, Providence, Rhode Island 02903, USA

## Abstract

Aspergillus endocarditis is an exceedingly rare entity associated with mortality rates of 60–80% even with combined surgical and medical therapy. It most commonly occurs in immunocompromised patients or those with prosthetic cardiac devices. We present a case with unusually extensive cardiac aspergillosis affecting multiple anatomical sites rarely seen simultaneously in a single patient.

Surgical management required radical debridement of infected tissue with subsequent reconstruction. This case uniquely illustrates the spectrum of pathologic involvement across multiple cardiac structures in a single patient, emphasizing both the diagnostic challenges and aggressive surgical approach necessary for survival.

## Background

Aspergillus endocarditis represents a rare but devastating infection, accounting for 0.2% of all infective endocarditis cases and approximately 25% of fungal endocarditis. Despite advances in antifungal therapy and surgical techniques, mortality remains exceptionally high at 60–80% even with aggressive treatment. Surgical debridement combined with antifungal therapy improves survival compared to medical management alone (34% versus 22%), though outcomes remain dismal.

Primary risk factors include solid organ transplantation with immunosuppression, hematologic malignancies, prosthetic valves, and indwelling cardiac devices. Diagnosis is challenging as blood cultures are positive in only 10% of cases, requiring high clinical suspicion and reliance on serum biomarkers (galactomannan, beta-D-glucan) or histopathological examination of surgical specimens.

Aspergillus endocarditis characteristically demonstrates extensive tissue invasion with abscess formation affecting multiple cardiac structures and high rates of systemic embolization (up to 70%), particularly to the central nervous system. Successful management requires radical surgical debridement, long-term antifungal therapy, and judicious immunosuppression modification.

We present a case of extensive native-valve Aspergillus endocarditis in a renal transplant recipient that uniquely illustrates the broad spectrum of cardiac involvement possible with this pathogen, including mitral valve vegetation, periaortic abscess, and septal involvement with ventricular wall extension—findings rarely documented together in a single patient.

### Case description & clinical images

A 68-year-old renal transplant recipient presented with acute left eye vision loss. The patient had experienced intermittent fevers for three weeks prior to presentation but had not sought medical attention during this period. Physical examination revealed left conjunctival hemorrhage. Laboratory studies demonstrated leukocytosis with marked neutrophilia (97%). Initial infectious workup was negative for cytomegalovirus and adenovirus.

**Figure 1.**
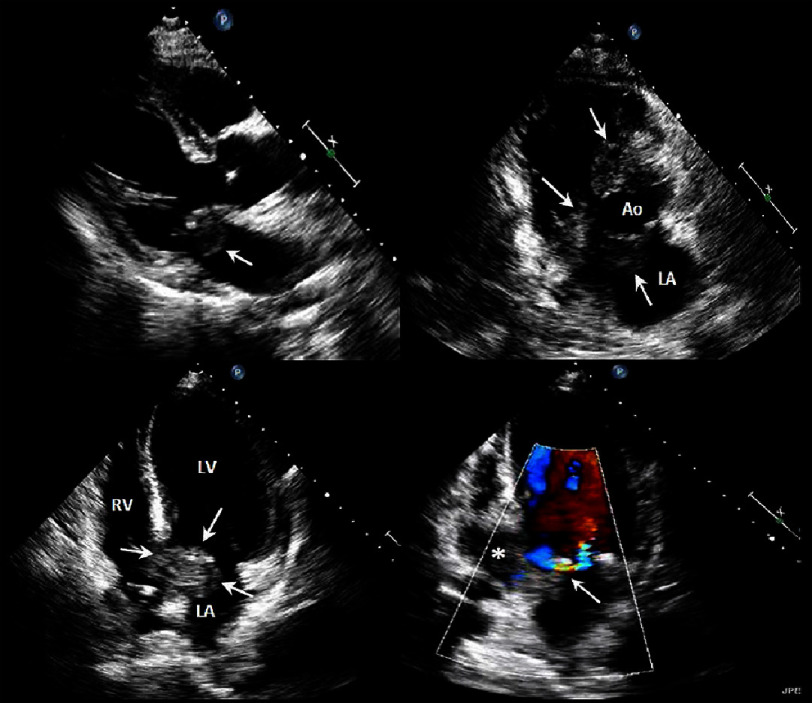
Transthoracic echocardiography. Demonstration of a mobile, invasive vegetation on the anterior leaflet of the mitral valve (Figure 1A, Video 1A, arrow). An abscess partially encircles the aorta (Figure 1B, Video 1B, arrows). The abscess involves both the interatrial and interventricular septa (Figure 1C, Video 1C, arrows). Figure 1D and Video 1D show the large abscess extending from the mitral valve annulus 4 centimeters into the inferior left ventricular wall (asterisk). Flow demonstrates a hole (Figure 1D, Video 1D, arrow) in the mitral valve leaflet.

Echocardiography (Figure 1, Videos 1A–D) revealed extensive cardiac involvement. A mobile vegetation was visualized on the anterior mitral leaflet (Figure 1A, Video 1A, arrow). A large periaortic abscess partially encircled the aortic root (Figure 1B, Video 1B, arrows) with extension into both the interatrial and interventricular septa (Figure 1C, Video 1C, arrows). The abscess extended four cm from the mitral annulus into the inferior left ventricular wall (Figure 1D, Video 1D, asterisk). Color Doppler imaging demonstrated a perforation through the mitral leaflet (Figure 1D, Video 1D, arrow).

**Figure 2.**
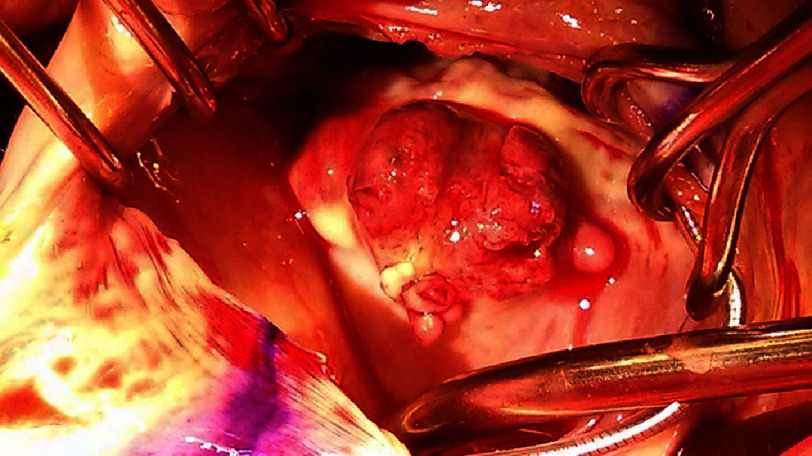
A large abscess which involved both septa as well as surrounding structures.

The patient underwent urgent cardiac surgery. Intraoperative findings (Figure 2) confirmed an extensive fungal abscess involving the interventricular and interatrial septa with invasion of adjacent cardiac structures. Radical debridement of infected tissue was performed, followed by septal reconstruction using bovine pericardium. Both mitral and tricuspid valves required excision with bioprosthetic replacement. Concomitant explantation of chronically implanted pacemaker leads (in situ for 4 years) was performed due to suspected device involvement.

**Figure 3.**
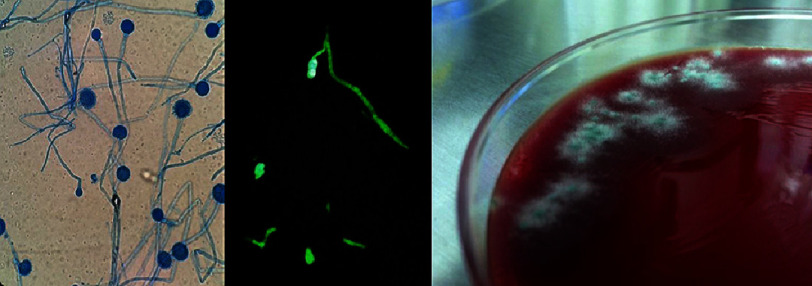
*Aspergillus fumigatus***culture**. Left - Grocott’s methenamine silver stain; middle –fluorescent stain, right - plate.

Surgical specimens cultured *Aspergillus fumigatus* (Figure 3). Antifungal susceptibility testing demonstrated sensitivity to both voriconazole and amphotericin B. Combination antifungal therapy was continued throughout hospitalization. The immunosuppressive regimen was modified: tacrolimus and prednisone were maintained to prevent allograft rejection, while mycophenolate mofetil was discontinued to enhance antifungal immunity.

The patient demonstrated favorable initial recovery over the first three postoperative weeks, progressing to the step-down unit with physical therapy and planned discharge. His course was subsequently complicated by acute mesenteric ischemia, suspected sepsis, thrombocytopenia, and microangiopathic hemolytic anemia consistent with thrombotic microangiopathy. Despite maximal supportive care, the patient’s condition deteriorated rapidly on postoperative day 21, necessitating reintubation and return to intensive care.

The family elected to transition to comfort measures on postoperative day 23, and the patient expired shortly thereafter. Autopsy was declined.

### What have we learned?

Aspergillus endocarditis carries exceptionally high mortality (60–80%) despite aggressive treatment. Surgical intervention significantly improves outcomes, with survival rates of approximately 34% versus 22% in medically managed cases. Without combined surgical and antifungal therapy, mortality exceeds 75%^1^.

This patient had multiple established risk factors for Aspergillus endocarditis, including solid organ transplantation with chronic immunosuppression^2–5^ and indwelling cardiac devices (chronically implanted pacemaker leads)^6,7^. Lead explantation was performed as an essential component of source control during surgical debridement.

This case demonstrates the broad spectrum of cardiac involvement possible with Aspergillus endocarditis, with multisite pathology documented in a single patient. Fungal infection affected the mitral valve apparatus, both atrial and ventricular myocardium, and extended circumferentially around the aortic root with perivalvular abscess formation. Despite this extensive cardiac involvement, successful surgical management required radical debridement and reconstruction of all infected structures to achieve source control.

Clinicians must maintain heightened suspicion for Aspergillus endocarditis in immunocompromised patients, particularly solid organ transplant recipients and those with indwelling cardiac devices. Early diagnosis is critical, as surgical intervention offers the only reasonable chance for survival^8–11^. Despite aggressive surgical and medical management, this patient succumbed to complications on postoperative day 23. While mortality remains high, combined surgical debridement and antifungal therapy represents the standard of care and should be pursued in surgical candidates with adequate functional reserve.
